# Relationships of beta-casein genetics with production, fertility, and survival of purebred organic Holstein dairy cows

**DOI:** 10.3168/jdsc.2022-0367

**Published:** 2023-09-22

**Authors:** S.C. Arens, K.T. Sharpe, M.M. Schutz, L.C. Hardie, C.C. Dechow, B.J. Heins

**Affiliations:** 1 Department of Animal Science, University of Minnesota, St. Paul, MN 55108; 2 Department of Animal Science, Pennsylvania State University, University Park, PA 16802

## Abstract

The objective of this study was to compare β-casein genotype of purebred certified-organic Holstein cows, and their effect on production, fertility, and survival. Holstein cows (n = 1,982) from 13 certified-organic dairy herds from the western, midwestern, and northeastern United States were genomically tested with CLARIFIDE Plus (Zoetis) for β-casein genotype. Two hundred fourteen cows were A1A1 (11%), 848 cows were A1A2 (43%), and 920 cows were A2A2 (46%). In total, 2,249 lactation records, 1,025 from the first parity and 1,224 records during second and greater parities were used. Test-day milk, fat, and protein production (305-d) and somatic cell score were obtained from the Dairy Herd Improvement Association. A lower limit of 50 d for days open was applied, and cows with more than 250 d open had days open set to 250 d. Independent variables for statistical analysis were the fixed effects of herd, parity, β-casein genotype (A1A1, A1A2, A2A2), and β-casein genotype by parity interaction. Cow nested within parity was the random effect in the statistical models for fertility and production traits. Herd had a significant effect on all fertility, production, and survival variables. Parity affected the number of times bred per pregnancy and days open, milk, fat, and protein production, and somatic cell score. Beta-casein genotype and herd influenced the percentage of cows surviving to first and second lactation. Results indicate no difference in production and fertility regarding β-casein genotype for organic dairy herds. Survival was biased against the A1 allele, which is indicated by lower survival rates during first lactation. These results may offer organic producers more flexibility in breeding and culling decisions to produce A2A2 milk.

## Highlights

• The frequency of the A2 allele (68%) was greater than the A1 allele and the A2/A2 genotype was most common (46%) for this population of organic Holsteins.
• Beta-casein genotype had no effect on days open or number of times bred.
• Beta-casein genotype had no effect on milk, fat, or protein yields or somatic cell score.
• Survival was affected by β-casein genotype, likely because farmers culled against the A1A1 genotype.

## Graphical Abstract

**Figure:**
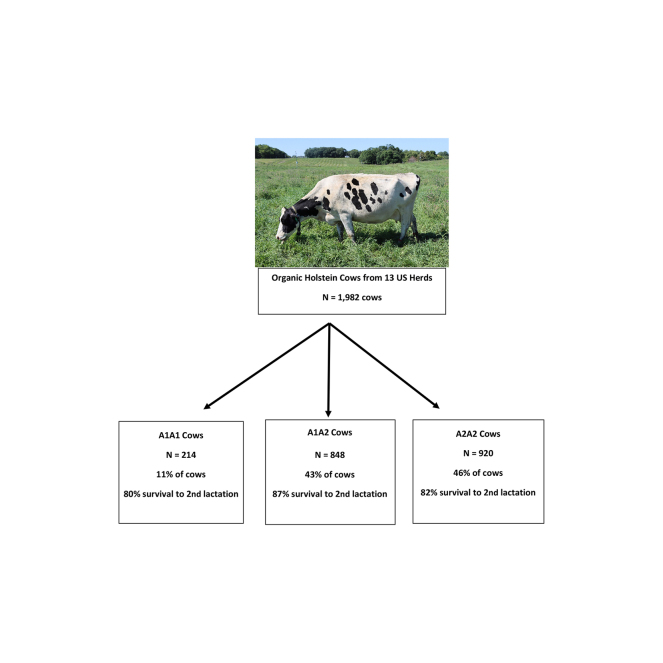
**Summary:** This study evaluated β-casein genetics of organic Holstein cows and its effect on production, fertility, and survival. Beta-casein genotype did not affect fertility or production. Herd and parity affected fertility and production more than β-casein genotype. The negative connotation with the A1 genotype might have resulted in premature culling of productive organic dairy cattle.

Protein constitutes 3.5% of milk components (Davoodi et al., 2016) and is imperative to development, maintenance, and repair of body tissues in addition to providing an energy source for cattle (Lu et al., 2020). Caseins and whey proteins account for 95% of protein in milk with the remaining 5% consisting of peptones, low molecular weight peptides, and fat globule membrane proteins (Sodhi et al., 2022). Caseins in milk are biologically found in 4 variants: α_S1_, α_S2_, β, and κ, and the frequencies at which these are found within a population have changed over time (Givens et al., 2013; Mayer et al., 2021; Park and Haenlein, 2021; Daniloski et al., 2022). Beta-casein accounts for one-third of milk casein content and consists of 12 genetic variants (Lambers et al., 2021; Bisutti et al., 2022). The most common forms of β-CN are A1 and A2 (Mayer et al., 2021; Daniloski et al., 2022). The A1 and A2 alleles are co-dominant and additive in their effect, and the frequency distribution of A1 and A2 alleles can vary by breed and geographic location (Woodford, 2007; Sodhi et al., 2022). Truswell (2005) reported the A1 allele is the most common allele found in dairy cattle of Northern European origin such as the Friesian, Holstein Friesian (**HO**), and Ayrshire versus the A2 allele, which is more commonly observed in the Channel Island breeds of Guernsey and Jersey (**JE**) cattle. The genotypic frequency of the A1 and A2 alleles may also vary and Bisutti et al. (2022) reported genotypic frequencies of 0.43 for A1A2, 0.33 for A2A2, and 0.14 for A1A1 and 0.10 for BA1 and BA2 from 1,133 Italian Holstein cows. The gene frequency of the A2 allele in US dairy cows is unknown because many cows are not tested for the A2 allele. However, according to the National Association of Animal Breeders Dairy Cross Reference Database, the frequency of the A2A2 genotype was over 43% for Holstein AI bulls (NAAB, 2023).

The A2 allele, historically, was the only predominant form of β-CN in genus *Bos*. However, a mutation occurred, which caused a histidine substitution at position 67 and consequently created the A1 variant. The A1 allele is less resistant to enzymatic digestion and subsequently releases β-casomorphin-7, whereas A2 does not cleave at position 67 and releases β-casomorphin-9 (Giglioti et al., 2020, 2021; Lambers et al., 2021; Sodhi et al., 2022). These β-casomorphins are considered opioid-like peptides and are found in human and bovine milk during digestion in the intestine and is suggested to negatively affect up to a quarter of the human population (Kaskous, 2020; Mayer et al., 2021).

Very few studies have evaluated β-CN genotypes and their effects on cow production and fertility. A New Zealand study in 2020 evaluated the effects of the A1 and A2 allele on cow production and fertility traits in HO, JE, and HO × JE crossbred cows (Lu et al., 2020). The study reported that β-CN genotype did not have a significant effect on fertility in cows. The β-CN genotype within lactation number had no effect on fertility; however, herd and β-CN genotype did have an effect on the pregnancy rate at 42 d after the start of the breeding season. Furthermore, β-CN genotype did not affect total milk, fat, or protein production. Conversely, Ng-Kwai-Hang et al. (1990) reported HO A2A2 cows had more milk production than A1A1 cows. Morris et al. (2005) reported that A2A2 cows had higher fat and protein production than A1A1 cows in New Zealand dairy cattle. Lu et al. (2020) concluded that the differences reported from research studies may be attributed to the potential of gene linkage because casein genes on BTA 6 are closely linked, consequently making it difficult to distinguish the difference between the effect of linked genes or the β-CN loci.

Interest in A2A2 milk is growing, and given the heightened health consciousness of organic dairy consumers, quantifying the frequency of the A2 allele in the US organic population is important. This may provide insight on how quickly the organic dairy industry could adapt to market shifts if demands for A2 milk continue to grow.

Many genomic testing panels include the marker that distinguishes between the A1 and A2 β-CN types. Most research with the A2 allele has focused on human health aspects; however, few studies have compared the effect of β-CN genotype on dairy cow production and fertility from the allele itself or other closely linked genes. Most studies investigating β-CN genotype in dairy cows have also been conducted on nonorganic dairy herds. Therefore, the objective of this study was to evaluate organic HO cow milk production, milk components, SCS, and fertility from cows genotyped for the A1 and A2 alleles.

All procedures involving animal care and management were approved by Penn State Institutional Animal Care and Use Committee (protocol #47560). Data were collected from HO cows on 13 USDA certified-organic dairy herds located in the northeastern, midwestern, and western portions of the United States (Hardie et al., 2021).

Holstein cows and heifers (n = 1,982) were genotyped with CLARIFIDE Plus from Zoetis (https://www2.zoetisus.com/solutions/dairy/dairy-genetics/) for β-CN genotype and had breeding events recorded from on-farm software. In total, 2,249 lactation records included 1,025 records from first parity and 1,224 records from second parity and greater. From the herds in the current study, 7 herds (n = 881 HO cows) had test-day milk records for milk, fat, and protein production and SCS from DHIA milk recording. Best prediction was used to calculate actual production (milk, fat, protein, and SCS) for 305-d lactations with adjustment for age at calving, and records less than 305 d were projected to 305 d (Cole et al., 2009). Herds were selected based on industry personnel feedback of herds who recorded production, fertility, and survival (Hardie et al., 2022). Data collected on-farm were from Dairy Comp305 (Valley Ag Software, Tulare, CA), PC Dart (Dairy Records management Systems, Raleigh, NC), DHI-Plus (Amelicor, Provo, UT), or handwritten records. Each herd was visited by researchers from 2017 to 2019 to collect supplemental data (Basiel et al., 2021; Hardie et al., 2021). Herds were variable in the accuracy of data recording and the amount of data provided for the study. Data were from birth years of animals from 2006 to 2018.

Fertility of cows included days open (**DO**), number of times bred per pregnancy (**TBRD**), and first-service conception rate (**FSCR**). The DO and TBRD were continuous variables and FSCR was recorded in a binary manner as either conceived or not conceived at first service. For DO, a lower limit of 50 d was applied and those with more than 250 d DO had DO set to 250 d. The maximum of 250 d for DO is used by the Council on Dairy Cattle Breeding for routine genetic evaluations for cow fertility in the United States. All 1,982 genotyped animals were used in the analysis for survival to first lactation (**SURV1**) and second lactation (**SURV2**), and were recorded in a binary manner as whether or not the animal survived to the respective lactation. The SURV1 was survival from live birth to first calving (Hardie et al., 2021). Production traits were 305-d milk, 305-d fat, 305-d protein, and SCS from DHIA milk recording. Animals without a β-CN genotype were not used in any analysis. Some cows were genotyped for β-CN genotype after first calving, which may have affected the outcome of variables analyzed in this study.

For the analysis of FSCR, DO, and TBRD, the fixed effects were herd, parity group (primiparous or multiparous), β-CN genotype, and the β-CN genotype by parity interaction with cow nested within parity as a random variable. Sire of animal was also included in all statistical models as a random effect. Data were analyzed with a mixed model using PROC MIXED of SAS 9.4 (SAS Institute Inc., Cary, NC). Logistic regression was with PROC GLIMMIX of SAS 9.4 to analyze SURV1 and SURV2. The fixed effects of herd and β-CN genotype were included in the model. The 305-d milk, fat, and protein production and SCS were analyzed with the fixed effects of herd, parity group, β-CN genotype, and the interaction between β-CN genotype and parity group, with cow nested within parity as a random variable. Mixed models performed with PROC MIXED of SAS were also used for the statistical analysis of all production traits. Results for all variables were reported as least squares means and significance was declared at *P* < 0.05.

Two hundred fourteen cows had β-CN genotypes of A1A1 (11%), 848 cows had A1A2 (43%), and 920 cows had A2A2 (46%), which corresponds to an allele frequency of 67.8% for A2. The genotypic frequencies of the cows in the current study were higher than those reported by Bisutti et al. (2022), and the A1A1 genotype was least frequent. The frequency of the A2A2 genotype in the current study suggests that there is selection for the A2 allele in organic herds.

Least squares means for fertility and survival by herd are in Table 1. The mean herd size of herds used in the study varied. Some herds had many other breeds or crossbreds, but the current study only used Holstein cows and heifers. Herd significantly (*P* < 0.0001) explained variation for all fertility and survival traits. The DO ranged from 115 to 250 d, which was similar to the mean DO of 135 d in the United States reported by Pszczola et al. (2009). The range for TBRD in the current study was 1.0 to 2.81. Pinedo and Velez (2019) reported TBRD ranged from 1.7 to 2.85, and average 2.00, which is similar to the average of 1.89 for the herds in the current study. First-service conception rate ranged from 31% to 60%, which is similar to the range reported by Grimard et al. (2006) of 25.9% to 63.6% for herds. Survival to first lactation was high for all herds with the lowest survival rate of 85% for herd K. The percentage of survival to second lactation was lower for all herds with the lowest survival rate of 62% for herd K.

**Table 1:** Least squares means and SEM for fertility indicator traits and production measurements of Holstein dairy cows by herd^1^

Herd	Mean herd size	DO (d)		TBRD (no.)		FSCR (%)		SURV1 (%)		SURV2 (%)		305-d milk (kg)		305-d fat (kg)		305-d protein (kg)		SCS	
		LSM	SE	LSM	SE	LSM	SE	LSM	SE	LSM	SE	LSM	SE	LSM	SE	LSM	SE	LSM	SE
No. of records used		2,249		2,062		1,733		1,982		1,982		1,774		1,774		1,774		1,774	
A	64	121cegh	7	1.71^a^	0.21	45ace	5.2	92^ab^	3.5	83acf	6.8								
B	67	116^fg^	6	1.50^a^	0.17	55abef	4.3	97^a^	3.0	92^ef^	3.5	9,851^a^	109	368^ag^	4	310^a^	3	2.68^a^	0.10
C	98	141^dh^	9	1.95^a^	0.29	51abef	6.8	97^a^	4.8	86^ac^	6.2								
D	363	115^ef^	5	2.48^bc^	0.18	37^c^	3.7	89^b^	2.3	69^bc^	5.1	8,763^bf^	77	349^b^	3	287^bf^	2	3.10^b^	0.07
E	42	132^dg^	8	1.82^a^	0.26			99^a^	4.8	93ade	5.6	6,302^cd^	180	254^cd^	7	206^cd^	5	2.33acd	0.17
F	21	125def	11	1.92ace	0.33			99^a^	5.6	88acde	9.3	6,246^d^	204	271^d^	8	220^d^	6	2.57acd	0.12
G	29	142^d^	8	1.56^a^	0.25			99^a^	4.2	85ade	6.7	7,295^e^	132	295^e^	5	239^e^	4	2.56^ac^	0.12
H	18	142^d^	7	2.73bef	0.31	38acd	7.8	99^a^	3.8	98^de^	2.0								
I	58	142^d^	7	2.10acf	0.28	65^f^	6.3	94^a^	2.6	85ade	4.5	8,632^f^	99	355^fg^	4	290^f^	3	2.30^cd^	0.09
J	558	134^d^	5	1.64^a^	0.14	50^ae^	3.6	91^a^	2.7	87ade	4.0	9,535^g^	83	361^g^	4	325^g^	2	2.24^d^	0.08
K	6,029	128^cd^	8	1.00^dg^	0.15	45^ac^	5.9	85^b^	2.3	62^b^	7.1								
L	1,414	149^b^	5	2.46^bh^	0.15	31^d^	3.8	93^a^	2.6	79^bc^	5.0								
M	154	250^a^	12	1.81acfgh	0.71	60^ef^	7.7	93^ab^	5.1	90ade	5.7								
All herds		136		1.67		50		94.6		80.2		8,796		344		293		2.56	

a–h Means within a column without common superscripts are different at *P* < 0.05.

1 Reported means and SE are based on lactation by genotype averages. Number of lactation records used: days open (DO), 2,249; milk, fat, protein, and SCS, 1,774. Number of animals analyzed: first-service conception rate (FSCR) and number of times bred per pregnancy (TBRD), 1,733; survival to first lactation (SURV1) and survival to second lactation (SURV2), 1,982.

Milk, fat, and protein production for herds are displayed in Table 1. Herd significantly (*P* < 0.0001) explained variation for the production traits. The 305-d milk production ranged from 6,246 to 9,851 kg, 305-d fat from 254 to 368 kg, and 305-d protein ranged from 206 to 325 kg. The average SCS ranged from 2.24 to 3.10. While there were significant differences among herds within each production trait, no single herd had the highest or lowest yields for 305-d milk, fat, and protein. Nehring et al. (2021) reported organic herds average 6,594 kg of milk, which was lower than the average production of the 13 herds in this study. However, Fernandes et al. (2021) reported higher production averages from 2 Texas organic herds of 10,897 kg of milk. The SCS of the herds in the current study was lower than those reported by Fernandes et al. in the 2 large organic herds in Texas. Differences for production among herds was most likely caused by differences in herd management practices such as breeding protocols, environment and weather, grazing time, health treatment protocols, percentage of DM from pasture during the grazing season, winter feed rations, or production goals.

Table 2 includes results for fertility and survival traits by each β-CN genotype. Beta-casein genotype had no significant effect on FSCR, TBRD, and DO, which was also reported by Lu et al. (2020). Genotype did significantly affect SURV1 and SURV2. Cows with the A1A1 genotype had lower (93% **±** 1.2, *P* = 0.01) percent SURV1 than cows with the A1A2 genotype (96% **±** 1.2). Perhaps, organic farmers were culling heifers with the A1A1 genotype before they reached their first lactation. The SURV2 was higher for cows with the A1A2 genotype (90% ± 1.8) compared with those with A1A1 (85% ± 3.3; *P* = 0.06) or A2A2 (85% ± 2.3; *P* = 0.012). Some farms may have used the β-CN genotype results to cull cows before second lactation, thus inflating the number culled. However, the SURV2 was similar for A1A1 and A2A2 cows. As the niche market for A2 milk becomes increasingly popular, the demand for A2 milk, milk that contains only the A2A2 genotype, has increased. Therefore, some producers may wish to make culling decisions based on this growing demand to meet the needs of the producer's milk cooperative. More than likely, once the herds received the β-CN genotype results from the genomic testing, the herds culled the A1A1 cows more heavily without regard to production or fertility. Recently, Scott et al. (2023) reported that A2A2 cows had lower fertility and survival because of increased selection and inbreeding in A2A2 cows.

**Table 2:** Least squares means and SEM for fertility, survival, and production measurements of organic Holstein dairy cows by β-CN genotype^1^

Variable	A1A1		A1A2		A2A2	
	LSM	SE	LSM	SE	LSM	SE
First-service conception rate (%)	45.5	4.5	50.2	2.4	47.4	2.3
No. of times bred per pregnancy	1.86	0.15	1.88	0.10	1.92	0.10
Days open (d)	145	5	138	3	140	3
Survival to first lactation (%)	93^a^	1.2	96^b^	1.2	95^ab^	1.2
Survival to second lactation (%)	85^a^	3.3	90^b^	1.8	85^ab^	2.3
305-d milk (kg)	8,175	116	8,089	64	8,003	63
305-d fat (kg)	327	5	321	3	318	3
305-d protein (kg)	269	3	269	2	267	2
SCS	2.58	0.10	2.54	0.06	2.54	0.06

a,b Means within a row without common superscripts are different at *P* < 0.05.

1 Reported means and SE are based on lactation by genotype averages.

Beta-casein genotype had no effect on 305-d milk, fat, or protein, or SCS as shown in Table 2. The A1A1 cows had numerically higher production than A1A2 and A2A2 cows for 305-d milk and 305-d fat. Dairy cow studies are inconsistent when reporting β-CN genotypes and production. Lu et al. (2020) found no difference for production and β-CN genotype for HO, JE, and HO × JE crossbred cows. Çardak (2005) also reported β-CN genotype did not affect daily milk and protein or fat and protein percentages for HO cows. However, fat production of HO cows was different by β-CN genotype and A1A1 cows had higher fat production than A1A2 and A2A2 cows (Çardak, 2005). Meanwhile, Morris et al. (2005) reported A2A2 HO cows were superior to A1A2 HO cows for fat production. Scott et al. (2023) reported A2A2 cows had an increase breeding value for protein production compared with A1A1 cows in Australia. These inconsistencies could be caused by the environment, feed rations, health treatment protocols or breeding protocols.

Results for the interaction β-CN genotypes and parity on fertility are in Table 3. Parity and genotype had no significant effect on FSCR. First-service conception rate was numerically higher for both primiparous and multiparous cows with the A1A2 genotype. Mean FSCR for primiparous cows was similar to Donovan et al. (2003) who reported a FSCR of 47.1%. Pinedo and Velez (2019) reported an average conception rate of 43.3% for US organic herds from Dairy Records Management System DHIA data from 2017 to 2019, which was lower than the average of 47.7% for the current study. Lu et al. (2020) reported a FSCR of 45.7% and 54% for second and later parity cows, respectively, which was similar to the FSCR for multiparous cows in the current study. Furthermore, the authors reported A2A2 cows had a numerically lower FSCR than A1A1 and A1A2 cows; however, β-CN genotype had no effect on FSCR (Lu et al., 2020). Parity had a significant (*P* < 0.01) effect on TBRD. The A1A2 primiparous cows numerically had the lowest TBRD compared with the other β-CN genotype cows in first lactation. Furthermore, the A1A1 multiparous cows had numerically the lowest TBRD compared with the other β-CN genotype multiparous cows. Gebeyehu et al. (2007) reported parity had a significant effect on number of services per conception. Parity had an effect on DO but β-CN genotype did not affect the DO of cows. The A1A1 primiparous and multiparous cows had the highest numerical DO. Quite possibly, the DO differences may be caused by farmers not choosing to mate the A1A1 cows, thus increasing the DO of those cows. The DO differences may be caused by environmental factors as suggested by the low heritability of fertility traits (Hardie et al., 2022). The current study was similar to Lu et al. (2020) who reported no difference for β-CN genotypes for DO of New Zealand HO, JE, and HO × JE crossbred dairy cows.

**Table 3:** Least squares means and SEM for fertility and production measurements of organic Holstein dairy cows by parity and β-CN genotype^1^

Variable	Primiparous						Multiparous					
	A1A1		A1A2		A2A2		A1A1		A1A2		A2A2	
	Mean	SE	Mean	SE	Mean	SE	Mean	SE	Mean	SE	Mean	SE
First-service conception rate (%)	42.6	5.7	48.8	3.2	45.9	3.1	48.4	6.5	51.7	2.9	48.9	2.9
No. of times bred per pregnancy	2.28	0.18	2.20	0.11	2.27	0.11	1.44	0.20	1.56	0.11	1.57	0.11
Days open (d)	141	6	134	4	136	3	149	7	143	3	145	3
305-d milk (kg)	7,399	147	7,379	76	7,248	73	8,952	146	8,799	75	8,759	75
305-d fat (kg)	291	6	286	3	283	3	362	6	356	3	354	3
305-d protein (kg)	243	4	244	2	242	2	295	4	294	2	293	2
SCS	2.30	0.12	2.25	0.07	2.31	0.06	2.86	0.13	2.77	0.06	2.78	0.07

^a,b^ Means within a row, within parity, without common superscripts are different at *P* < 0.05.

1 Reported means and SE are based on lactation by genotype averages.

Table 3 also contains least squares means for 305-d milk, fat, and protein production and SCS by the β-CN genotype and parity interaction. Parity had a significant (*P* < 0.001) effect on all production traits; however, the interaction between β-CN genotype and parity did not affect production. Lu et al. (2020) also reported parity had a significant effect (*P* < 0.05) on milk, fat, and protein production for multiparous HO, JE, and HO × JE cows, but the interaction between parity and β-CN genotype was not significant with production. Conversely, Ng-Kwai-Hang et al. (1990) reported HO A2A2 cows had higher milk production than A1A1 cows during the first through third lactation, whereas the A1A2 cows were intermediate for milk production.

The results of the current study suggest that organic dairy farmers were selecting for the A2 allele. The A2 allele frequency in these herds was over 65%, suggesting that the A2 percentage has increased in US dairy herds across the last 10 to 15 yr. Because of the interest of farmers and consumers in A2A2 dairy products, the demand continues to increase for AI bulls with the A2A2 genotype from all breeds. Scott et al. (2023) reported that the A2A2 genotype increase by over 20% in a 17-yr period in Australia. Based on the Australian results, the A2A2 genotype in the United States would have been expected to increase by at least 20% over the past 20 yr as well.

This current study offered a unique perspective into certified-organic dairy farming in the United States and how genomic testing may affect herd management decisions. The results from this study found that the A2 allele is more common than the A1 allele in organic Holsteins. Fertility and production was not affected by β-CN genotype or by the β-CN genotype interaction with parity of organically raised cows and heifers. However, β-CN genotype was an important factor in survival and farmers may have used β-CN genotype as a factor when culling animals. The high survival rate of A2A2 animals was the result of selective culling of A1A1 or A1A2 genotypes by farmers. As the niche market for A2A2 milk continues to increase, more producers will be pushed to produce A2A2 milk to meet this new demand. However, most milk processors are not offering incentive payments for A2A2 milk. In the future, as consumers demand more from dairy products, processors may offer incentives to farmers that can prove they have a 100% A2A2-tested herd. Some farms may establish on-farm processing with A2A2 milk and sell it directly to the consumer, and this has been a growing opportunity in the United States. These might increase demand for confirmed A2A2 cows, and these cows may have increased value compared with A1A1 or A1A2 cattle. This study of over 2,000 animals showed that cows had similar production and fertility across β-CN genotypes. However, the negative connotation with the A1 allele might have resulted in premature culling of productive organic dairy cattle. Further research should focus on a larger number of cows to assess the impact of selecting on A2 β-CN genotype for productive and reproductive performance of dairy cattle in both organic and nonorganic dairy farms.
